# Genome-Wide Analysis of *ZAT* Gene Family in *Osmanthus fragrans* and the Function Exploration of *OfZAT35* in Cold Stress

**DOI:** 10.3390/plants12122346

**Published:** 2023-06-16

**Authors:** Huifen Ding, Zhandong Yang, Zhouying Zai, Keyi Feng, Lianggui Wang, Yuanzheng Yue, Xiulian Yang

**Affiliations:** 1 Key Laboratory of Landscape Architecture, Jiangsu Province, College of Landscape Architecture, Nanjing Forestry University, Nanjing 210037, China; 2 Co-Innovation Center for Sustainable Forestry in Southern China, Nanjing Forestry University, Nanjing 210037, China

**Keywords:** *Osmanthus fragrans*, zinc finger protein, cold stress, salt stress

## Abstract

*Osmanthus fragrans* is a popular ornamental and odorant plant with high commercial value, but its cultivation and exploitation are limited by low temperature. The *ZAT* (zinc finger of *Arabidopsis thaliana*) genes as a subclass of the C2H2-type zinc finger proteins (C2H2-ZFP) family play essential roles in various abiotic stresses. However, their roles in cold stress response in *O. fragrans* remain unclear. This study identified 38 *OfZATs*, which could be divided into 5 subgroups based on the phylogenetic tree, with *OfZATs* in the same subgroup harboring similar gene structures and motif patterns. In addition, 49 segmental and 5 tandem duplication events were detected among *OfZAT* genes, while some *OfZAT* genes exhibited specific expression patterns in different tissues. Furthermore, two *OfZAT*s were induced in salt stress and eight *OfZATs* responded to cold stress. Interestingly, *OfZAT35* showed a continuously increasing expression trend under cold stress, while its protein showed nucleus localization with no transcriptional activation activity. Transiently transformed tobacco overexpressing *OfZAT35* exhibited a significantly higher relative electrolyte leakage (REL) level and increased activities of superoxide dismutase (SOD), peroxidase (POD), and Ascorbate peroxidase (APX), while there was significantly decreased activity of catalase (CAT). Moreover, *CAT*, *DREB3*, and *LEA5*, which are associated with cold stress, were dramatically decreased after cold treatment in transiently transformed tobacco, suggesting that overexpression of *OfZAT35* negatively regulated cold stress. This study provides a basis for exploring the roles of *ZAT* genes and contributes to uncovering the mechanism of *ZAT*-mediated cold stress response in *O. fragrans*.

## 1. Introduction

*Osmanthus fragrans* is an ever-green, small ornamental tree or shrub, which is famous for its fragrant flowers and high commercial value. The *O. fragrans* flowers can be processed into food additives in pastries and drinks, such as tea, while its flower extracts are known to produce one of the best natural fragrant essences [1]. Due to its commercial benefits, the color and flower fragrance have become trending research topics in *O. fragrans* [2,3,4]. *Osmanthus* mostly grows in the warm temperature region of Asia [5]. The cultivation and commercial exploitation of the species *O. fragrans* is however limited by low temperature [6]. Therefore, low temperature is a key factor in osmanthus breeding. Several molecular studies on the *O. fragrans* response to cold stress have recently been reported. The genes of the bHLH transcription factor (TF) in *O. fragrans* induced by cold stress were screened through quantitative real-time PCR (qRT-PCR), while the functions of the NAC TF family in response to cold stress in *O. fragrans* were also screened using qRT-PCR [7,8]. The overexpression of an *O. fragrans* heat shock factor OfHSF11 in tobacco (*Nicotiana benthamiana*) under cold stress could negatively impact transgenic plant responses to cold treatment [9]. However, the molecular mechanism of sweet osmanthus response to low temperature stress is still unclear.

TFs are genes that regulate signal transduction and gene transcription, and their regulatory activities are associated with plant abiotic stress responses, such as cold stress [10]. The C2H2 zinc finger proteins (ZFPs) TF family was divided into many subfamilies, among which the C1 family is one of the largest subfamilies [11]. The members of the C1 family have different numbers of dispersed zinc fingers and were classed into five subclasses, including C1-1i, C1-2i, C1-3i, C1-4i, and C1-5i [11,12,13]. *ZAT* genes code the proteins which contain two dispersed zinc fingers and constitute the subclass C1-2i of the C2H2-ZFP TF family. [11,12]. Furthermore, most members of the ZAT family also have a highly conserved QALGGH motif and an EAR motif at the C-terminus [12]. To date, the systematic identification of members in the ZAT family has been performed in numerous plants, such as *Petunia hybrida*, *A. thaliana*, *Triticum aestivum*, *Gossypium hirsutum*, *Fragaria* × *ananassa*, and *Populus trichocarpa* [11,13,14,15,16,17]. However, despite their potential crucial roles in cold stress response, members of the *ZAT* gene family have yet to be characterized in *O. fragrans*.

The roles of *ZAT* genes have been reported in responding to cold stress and other abiotic stresses, with some members playing crucial regulatory roles in cold stress pathways [18]. Transcriptomic analyses have shown that the *ZAT* genes are significantly differentially expressed under cold stress in herbaceous plants, such as *Vicia sativa*, *Nicotiana tabacum*, and *Brassica napus*, as well as woody plants, such as *Taxillus chinensis*, *Citrus reticulata* ‘Chongyi’, and *Jatropha curcas* [19,20,21,22,23,24]. Studies on the *ZAT* gene family responses to cold and other stresses have extensively been reported in *Arabidopsis*. For example, the *Arabidopsis ZAT6*, *ZAT7*, *ZAT10*, and *AZF2* genes have been shown to be responsive to cold, dehydration, and high-salt stress, with some enhancing cold stress tolerance in the plant [25,26,27]. The cold responsive regulatory mechanism of *AtZAT*s has also been reported. The cold stress-responsive *RD29A* gene is a target of *CBF*, and its transcription is repressed by *AtZAT10*, leading to enhanced cold tolerance [28]. In addition, *AtZAT12* could affect cold tolerance in *Arabidopsis* by directly repressing *CBF* and also through the regulation of non-*CBF* signaling pathway genes during cold stress [29,30,31]. 

In this study, the published genome-wide sequence and transcriptome of *O. fragrans* were screened to characterize the *ZAT* gene family and to explore their expression profiles in various organs or tissues and under cold and salt stresses [4,9,32]. As a result, 38 *ZAT* genes were identified. Phylogenetic classification, duplication events, subcellular localization prediction, gene structure analysis, and expression patterns in various organs or tissues and under cold and salt stresses were performed. Based on gene expression patterns, a candidate *OfZAT35* gene was selected and transiently overexpressed in tobacco to investigate its likely function in cold stress. Our works not only provide a foundation for the functional evaluation of *ZAT* genes, but also contribute to uncovering the molecular regulatory mechanism of cold stress response in *O. fragrans*.

## 2. Results

### 2.1. Identification, Distribution, Duplication, and Physicochemical Characteristics of OfZATs

**Figure 1:**
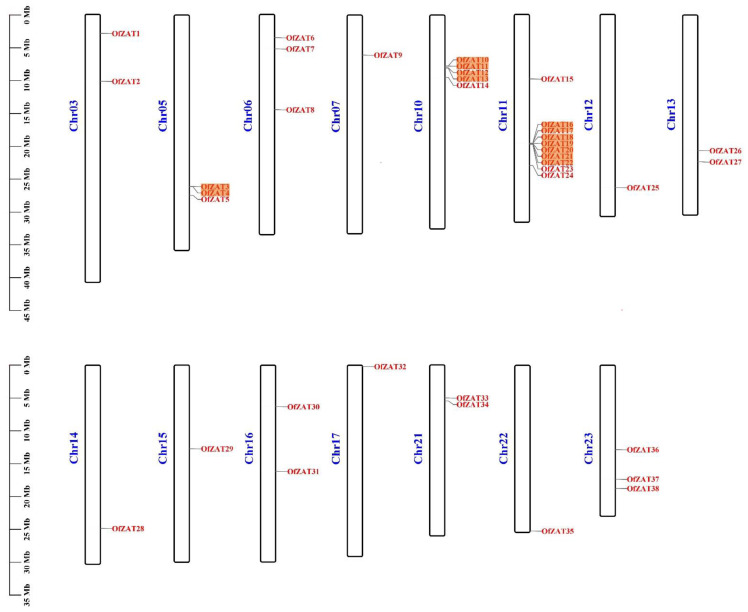
Chromosome distribution of *OfZAT* genes in the *O. fragrans* genome. The serial numbers of chromosomes are listed in blue on the left of each chromosome. The names of genes are listed on the right of each chromosome in red, and the genes with orange background originated from tandem duplication.

Thirty-eight candidate *ZAT* genes identified using HMMER 3.0 and tagged as *OfZAT1*–*38* based on their chromosomal locations were identified in the *O. fragrans* genome (Figure 1). Except for chromosomes (Chr) 1, 2, 4, 8, 9, 18, 19, and 20, *ZAT* genes were unevenly scattered across 15 *O. fragrans* chromosomes, with the highest density of 10 genes being observed in Chr 11, while other chromosomes contained 1–5 *ZAT* gene members.

The analysis of *ZAT* gene duplication events using the MCScanX program identified 49 segmental and 5 tandem duplication events in the *O. fragrans* genome (Figure S1). The 5 tandem duplication events occurred in 13 genes, including *OfZAT3*/*4*, *OfZAT10*/*11*, *OfZAT12*/*13*, *OfZAT16*/*17*, and *OfZAT18*/*19*/*20*/*21*/*22*, which were detected in Chr 5, Chr 10, and Chr 11. Additionally, 49 segmental duplication events were observed in 27 genes, which demonstrated that the *ZAT* gene family predominantly originated from segmental and tandem duplication events.

The physicochemical evaluation of OfZATs revealed that the predicted protein molecular weight (MW) ranged from 19.50 to 55.83 kDa in OfZAT13 and OfZAT37, respectively, while the isoelectric point (pI) varied from 6.11 in OfZAT17 to 9.73 in OfZAT4. Moreover, the prediction of subcellular localization exhibited that all predicted OfZATs are located in the nucleus (Table S1).

### 2.2. Phylogenetic Analysis of ZAT Genes

**Figure 2:**
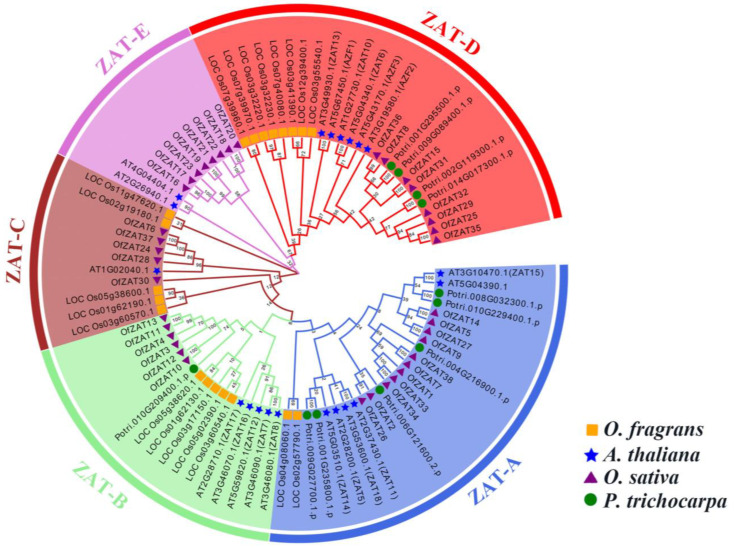
Evolutionary relationship of *ZAT* genes in sweet osmanthus (*O. fragrans*), Arabidopsis (*A. thaliana*), rice (*O. sativa*), and black cottonwood (*P. trichocarpa*) constructed using the NJ method. The values indicated 1000 bootstrap replication supports. The *ZAT* genes were classified into five subgroups that are highlighted in different colors.

The 38 predicted *OfZATs* along with 20, 20, and 11 homologous genes retrieved from Arabidopsis (*A. thaliana*), rice (*Oryza sativa*), and black cottonwood (*P. trichocarpa*), respectively, were used for phylogenetic tree construction with the neighbor-joining (NJ) method to explore their evolutionary relationships. According to the types of motifs (Figure S2), the 89 genes could be clustered into 5 subgroups, including ZAT-A, ZAT-B, ZAT-C, ZAT-D, and ZAT-E (Figure 2). The sequences of motifs are displayed in Table S2. Notably, ZAT-A was the largest subgroup containing 25 genes, while ZAT-C was the smallest subgroup with 11 members.

### 2.3. Structure and Motif Composition of OfZATs

**Figure 3:**
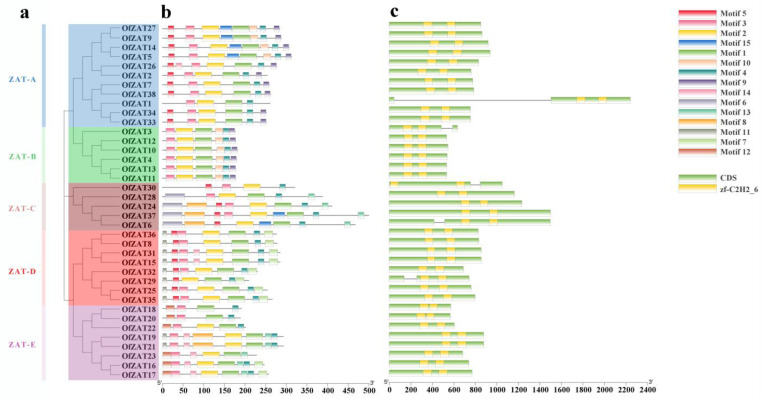
The phylogenetic classification, gene structure, and motif composition of *OfZATs*. (**a**) The phylogenetic trees of *OfZATs* showing different subgroups are marked with different colors. (**b**) Conserved motifs in *OfZATs* are highlighted in different colors. (**c**) The gene structures of *OfZATs* showing introns and exons. The yellow, green, and grey line regions represent conserved domains, CDS, and introns, respectively.

To further analyze the evolutionary relationships among *OfZAT*s, their structural and motif composition were analyzed (Figure 3). A varied number of exon–intron distributions were observed among *OfZAT* genes. Interestingly, introns were absent in most *OfZAT*s, with only five genes (*OfZAT1*/*3*/*6*/*29*/*30*) having one intron in their sequences. Motif identification using the MEME tool revealed 15 conserved OfZATs motifs, with OfZAT proteins in the same subgroup harboring similar motifs in both position and type. For example, members of the ZAT-B subgroup all contained Motifs 3, 2, 1, 10, 4, and 9 with similar permutations, while most members of the ZAT-A subgroup contained Motifs 5, 3, 2, 1, 4, and 9 with a similar permutation. Notably, Motif 5 was only absent in OfZAT1. In addition, most OfZATs contained Motif 1, Motif 2, and Motif 3. Motif 1 contains the conserved sequence CX_2_CX_3_FX_2_GQALGGHX_3_H, which was reported as first zinc finger domain, and the conserved sequence CX_2_CX_3_FX_3_QALGGHX_3_H in Motif 2 (Table S3) was reported as the second zinc finger domain (X represents arbitrary amino acid, and the number represents the quantity of amino acid) [18]. Motif 3 contains the conserved sequence AX_2_LX_2_L (Table S3), while the conserved sequence has not been reported. The sequences of the 15 motifs are displayed in Table S3.

### 2.4. Cis-Elements Analysis in OfZATs

The promoter screening of *OfZATs* was performed to facilitate the prediction of their potential biological functions. After excluding the universal, incomplete, and unannotated *cis*-elements, a total of 32 *cis*-elements, which were associated with four categories, including hormones, stress, development, and light responses, were identified (Figure S3). The hormone category contained 11 *cis*-elements, which were associated with the regulation of abscisic acid (ABA), methyl-jasmonate (MeJA), gibberellin (GA), Auxin, and salicylic acid (SA). Of the 38 predicted *OfZATs*, 32 (84.21%) contained 109 ABRE *cis*-elements, which are associated with the ABA response pathway, while 30 *OfZATs* (78.95%) contained 76 CGTCA and 76 TGACG-motifs, which are related to MeJA regulation. The development and light categories contained 8 *cis*-elements, and 31 genes (81.58%) harbored 109 G-boxes that are associated with light response. The stress group contained six *cis*-elements related to anaerobic induction, drought, wound, and low temperature stress. These results suggested that most members of the ZAT family in *O. fragrans* might potentially be involved in the ABA, MeJA, and light response processes, which provide a reference for further investigation.

### 2.5. Expression Pattern of OfZAT Genes in Different O. fragrans Tissues

**Figure 4:**
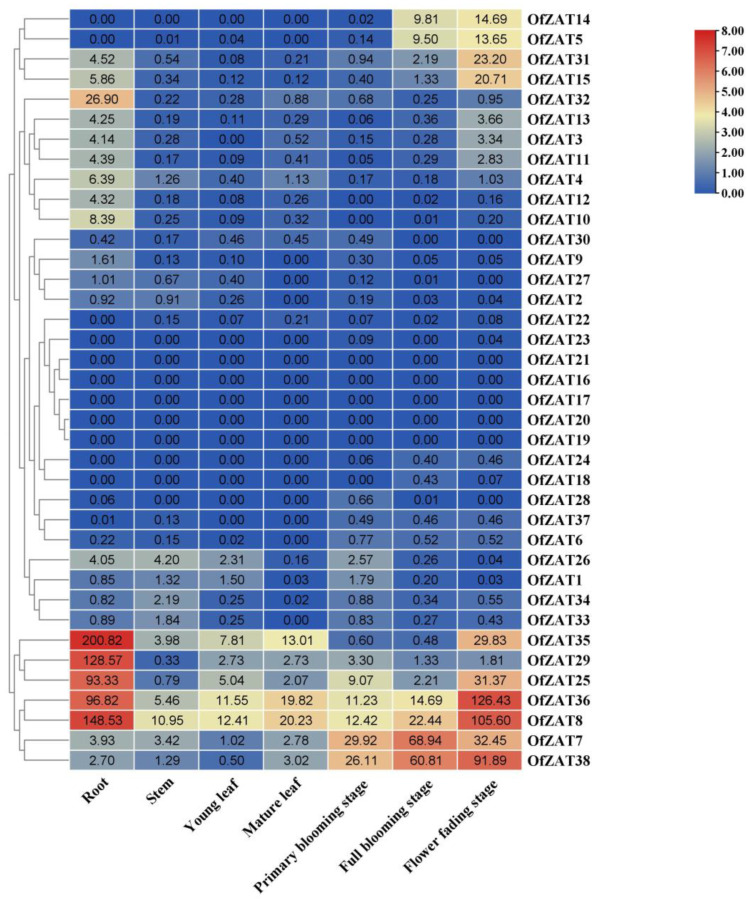
The temporal and spatial expression profiles of *OfZATs* in different *O. fragrans* tissues. The hierarchically clustered heat map was constructed using the FPKM values from transcriptome data converted to log_2_ (FPKM value + 1). The original FPKM values are shown in the heat map. The column legend on the right stands for the color of log_2_ (FPKM value + 1) in the heat map.

The fragments per kilobase million (FPKM) values of *OfZAT*s in four tissues, including the root, stem, leaves, and flowers, were obtained from previously reported transcriptome data (Table S4) which have been published in NCBI (SRP143423) [4]. The heat map was generated using TBtools according to the FPKM values from the transcriptome (Figure 4). Generally, FPKM values between 0 and 1 were considered as low expression levels [33]. Here, nine genes (*OfZAT2*/*6*/*18*/*22*/*23*/*24*/*28*/*30*/*37)* with low expression profiles were detected, while five genes (*OfZAT16*/*17*/*19*/*20*/*21*) were not expressed in all tissues (FPKM = 0) (Figure 4). In the root, there were seven *OfZATs* (*OfZAT8*/*15*/*25*/*29*/*32*/*35*/*36*) expressed highly (FPKM > 10), among which the expression level of *OfZAT35* was the highest (FPKM = 200.82). In the stem, most *OfZATs* were not expressed or poorly expressed, and only *OfZAT8* was expressed. In the leaf (both young and mature), only two *OfZATs* (*OfZAT8*/*36*) were expressed highly. In the flower, there were 10 *OfZATs* (*OfZAT5*/*7*/*8*/*14*/*15*/*25*/*31*/*35*/*36*/*38*) expressed highly. *OfZAT7* and *OfZAT38* exhibited the highest expression level in the full blooming stage among three flowering stages. However, the expression levels of eight *OfZATs* (*OfZAT5*/*8*/*14*/*15*/*25*/*31*/*35*/*36*) were highest at the flower fading stage during three flowering stages. Numerous genes have been shown to exhibit unique expression profiles in specific tissues, and the observed preferential tissue specific expression of *OfZATs* suggested their broad participation in plant growth and development.

### 2.6. Expression Patterns of OfZATs under Salt Stress

**Figure 5:**
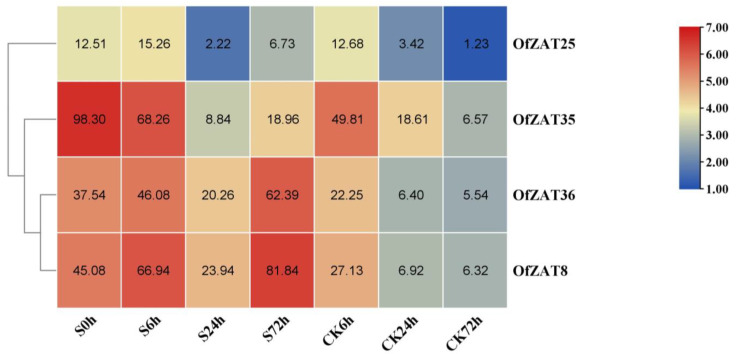
The expression profiles of four OfZATs (OfZAT8/25/35/36) in the salt stress treatment (S0 h, S6 h, S24 h, S72 h) and control (S0 h, CK6 h, CK24 h, CK72 h). The hierarchically clustered heat map was constructed using the FPKM values converted to log_2_ (FPKM value + 1). The original FPKM values are shown in the heat map. The column legend on the right stands for the color of log_2_ (FPKM value + 1) in the heat map.

For salt treatment, the seedlings were planted in 1/2 Hoagland’s nutrient solution with 250 mM NaCl solution and set at four time points (S0, S6, S24, and S72 h) to collect samples for transcriptome sequencing. In addition, the seedlings of the control group for salt stress were soaked in 1/2 Hoagland nutrient solution and also set at four time points (S0, CK6, CK24, and CK72 h) to collect samples for transcriptome sequencing. The values of FPKM were extracted from unpublished transcriptome data (Table S5) and submitted to TBtools to generate the heat map (Figure S4). As a result, four genes (*OfZAT8*/*25*/*35*/*36*) showed higher expression levels (FPKM > 10), while the remaining exhibited low (FPKM < 1) or no expression (FPKM = 0) under salt stress (Figure S4). The expression trends of *OfZAT25* and *OfZAT35* were decreased overall during salt treatment. However, the expression trends of *OfZAT25* and *OfZAT35* were also decreased in the control. In contrast, the levels of *OfZAT8* and *OfZAT36* exhibited an increasing trend under salt treatment, but with a decreasing pattern in the control treatment, which suggested that *OfZAT8* and *OfZAT36* play pivotal roles in salt stress response (Figure 5). 

### 2.7. Expression Patterns of OfZATs under Cold Stress

**Figure 6:**
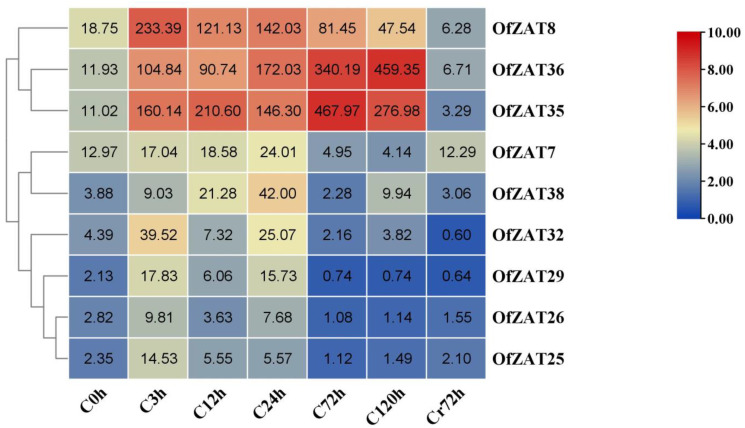
The expression profiles of nine *OfZATs* (*OfZAT7*/*8*/*25*/*26*/*29*/*32*/*35*/*36*/*38*) during cold stress treatment. The six periods during cold treatment were represented as C0 h, C3 h, C12 h, C24 h, C72 h, and C120 h. Cr72 h represented recovering 72 h after cold treatment. The hierarchically clustered heat map was constructed using the FPKM values converted to log_2_ (FPKM value + 1). The original FPKM values are shown in the heat map. The column legend on the right stands for the color of log_2_ (FPKM value + 1) in the heat map.

**Figure 7:**
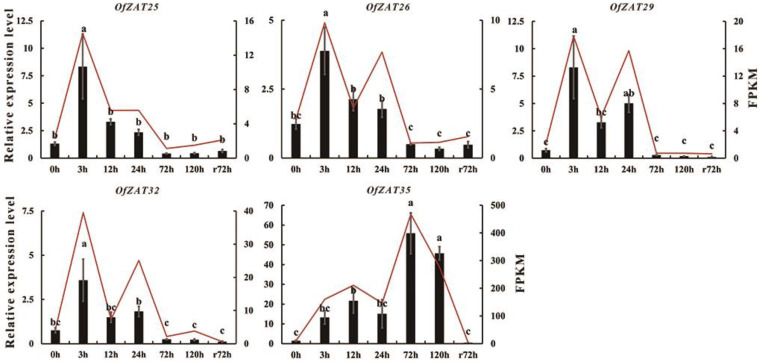
The relative expression levels of five selected *OfZATs* during cold treatment. The histograms indicating the data from qRT-PCR and FPKM values from transcription data are marked by a red line. The qRT-PCR data were statistically assessed using one-way ANOVA followed by Duncan’s test (*p* < 0.05), and the error bar represents ± SE (standard error) (*n* = 3). The letters (a, b, c) indicated the statistically differences based on Duncan’s test (*p* < 0.05).

During the 4 °C cold treatment, the samples were collected at seven timepoints (C0, C3, C12, C24, C72, and C120 h during cold treatment, and after recovery Cr72 h) for transcriptome sequencing. The values of FPKM were extracted from unpublished transcriptome data (Table S6) and submitted to TBtools to generate the heat map. Strong expression profiles (FPKM > 10) were only observed in eight *OfZAT* genes under cold treatment. In contrast, 29 genes (76.32%) were unresponsive to cold stress (FPKM ≤ 1), of which 10 genes (26.32%) were not expressed (FPKM = 0) in all stages (Figure S5). Under cold treatment, *OfZAT7* and *OfZAT38* were only upregulated at 0–3 h, and then downregulated in later stages. Five genes, including *OfZAT8*/*25*/*26*/*29*/*32*, were continuously upregulated 0–24 h before their overall expression levels decreased in the later periods. Both *OfZAT35* and *OfZAT36* were dramatically upregulated 0–72 or 0–120 h during cold treatment, and then decreased to a pre-treatment (0 h) level (Figure 6). Subsequently, qRT-PCR analysis was performed in five genes (*OfZAT25*/*26*/*29*/*32*/*35*). (Figure 7). As a result, consistent expression patterns were observed between the selected genes and those of transcriptome data in all stages, which demonstrated the reliability of RNA-seq data. Based on its unique expression level dynamics at different intervals after stress exposure, the *OfZAT35* gene was selected as a candidate for further investigation.

### 2.8. Subcellular Localization and Transcriptional Activation Activity 

**Figure 8:**
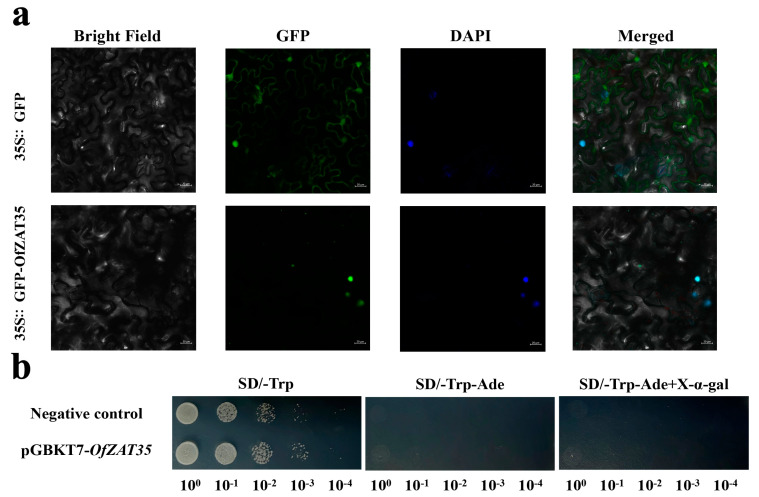
Subcellular localization assay and transcriptional activation activity of *OfZAT35*. (**a**) The subcellular location of *OfZAT35*. DAPI (4′,6-diamidino-2-phenylindole) was utilized to mark the nuclei florescent signals in the epidermal cells of tobacco. (**b**) The transcriptional activation activity of OfZAT35. The empty PGBKT7 vector (negative control) and recombinant plasmids pGBKT7-*OfZAT35* were transformed into yeast strain AH109. The yeast strain of negative control and *OfZAT35* (pGBKT7-*OfZAT35*) grew on different screening culture media (SD/-Trp, SD/-Trp-Ade, and SD/-Trp-Ade + X-α-gal medium). The number of 10^0^, 10^−1^, 10^−2^, 10^−3^, and 10^−4^ represents the concentration of dilution ratio of original yeast culture.

The coding sequence of *OfZAT35* was amplified and fused into 35S::GFP plasmids. The empty 35S::GFP vector and 35S::GFP-*OfZAT35* vector were transfected into leaves of 30 d tobacco (*N*. *benthamiana*), respectively. The fluorescent signals from the leaves revealed that the *OfZAT35* protein was only located in the nucleus, indicating its involvement in nucleus functions (Figure 8a). Moreover, pGBKT7-*OfZAT35* was also constructed using homologous recombination and then introduced to the AH109 yeast strain for a transcriptional activation activity assay. The yeast strains of the negative control (empty vector pGBKT7) and recombinant plasmids pGBKT7-*OfZAT35* grew in a different element deficient culture medium of SD/-Trp, SD/-Trp-Ade, and SD/-Trp-Ade + X-α-gal medium. *OfZAT35* and the negative control grew well in SD/-Trp medium, but not in other media, which indicated the absence of its transcriptional activation activity (Figure 8b).

### 2.9. Analysis of Physiological Parameters

**Figure 9:**
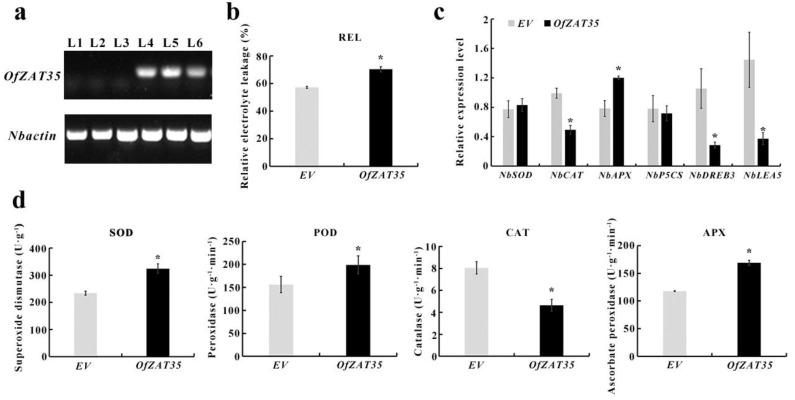
Physiological and biochemical analysis of transiently transformed tobacco. The data were statistically assessed using Duncan’s test (* *p* < 0.05). (**a**) The sqRT-PCR analysis of transiently transformed tobacco of *OfZAT35* and EV. The *Nbactin* was selected as a reference gene. L1−3 represent three transiently transformed tobacco lines of EV, while L4−6 represent transiently transformed lines of *OfZAT35*. (**b**) The analysis of REL in cold stress treated tobacco. (**c**) qRT-PCR analysis of ROS (reactive oxygen species) and cold-stress-related genes. (**d**) Analysis of SOD, POD, CAT, and APX antioxidant enzyme activities.

The fused Super1300-*OfZAT35* plasmids and empty Super1300 vector (EV) were transfected into leaves of 30 d tobacco (*N*. *benthamiana*) by an Agrobacterium-mediated transient expression method. Then, the transiently transformed tobaccos of *OfZAT35* and EV were treated for 6 hours under 4 °C. The *OfZAT35* was overexpressed according to the result of semi-quantitative RT PCR (sqRT-PCR) (Figure 9a). After 6 h of cold treatment at 4 °C, the relative electrolyte leakage (REL) of transiently transformed tobacco was significantly increased compared to that of the empty vector (EV) (Figure 9b), and the REL level is widely used as an indicator of cell membrane damage [34,35]. Thus, we speculated that the transiently transformed tobacco suffered more intense stress than the EV after 6 h cold treatment at 4 °C.

The activities of superoxide dismutase (SOD), peroxidase (POD), and Ascorbate peroxidase (APX) were significantly increased in the transiently transformed tobacco compared to the EV under cold stress (Figure 9d). However, the activity of catalase (CAT) was significantly reduced (Figure 9d). Notably, the expression levels of *NbAPX* and *NbCAT* were consistent with the activities of APX and CAT, respectively (Figure 9c). To analyze the response mechanism of transiently transformed tobacco to cold stress, the expression profiles of cold-related genes were determined using qPCR. The results showed that the levels of *NbDREB3* and *NbLEA5* were significantly decreased (Figure 9c). Overall, transiently transformed tobacco overexpressing *OfZAT35* displayed more cold stress effects than EV plants.

## 3. Discussion

In this study, 38 members of the ZAT family in *O. fragrans* were identified, and the number is higher than the number reported in the herbaceous *A. thaliana* (20) and *O. sativa* (20) plants or in the woody plant *P. trichocarpa* (11) [11,14,36]. Synteny analysis detected 49 segmental and 5 tandem duplication events in the *OfZAT* genes (Figure S1). Large genomic duplication events are known to drive the evolution and expansion of gene families [37,38,39]. For example, the *A. thaliana* genome has undergone at least 4 major duplication events between 100 and 200 million years ago (MYA), with segmental and tandem duplication contributing to the generation and maintenance of gene families [37,40]. Correspondingly, the *O. fragrans* genome has experienced 2 duplication events that occurred approximately 14 MYA, which might have contributed to the larger size of the *OfZAT* gene family [4].

The 38 candidate *OfZAT* genes were divided into 5 subgroups, and the genes within the same subgroup displayed similar motif types and arrangement, indicating that *OfZATs* in the same subgroup may have a similar function, and the classification of *OfZAT*s was reliable (Figure 3). Motif 1, Motif 2, and Motif 3 are present in most OfZAT members, and Motif 1, Motif 2, and Motif 3 were also found in most ZAT proteins of *A. thaliana*, *O. sativa*, and *P. trichocarpa* (Figure S2). Similarly, Motif 1, Motif 2, and Motif 3 have been reported in most members of the ZAT protein family in herbaceous plants, such as *Fragaria* × *ananassa* [17]. This indicated that the three ZAT motifs might be conserved in different plant species, and their similar clustering in same subgroup demonstrated the reliability of phylogenetic tree clusters. The structural analysis revealed the absence of introns in most *OfZAT* genes (Figure 3c), which was consistent with the observations made in *G. hirsutum* and *P. trichocarpa* [14,16]. Introns are predicted to delay regulatory responses, and genes with fewer introns are rapidly activated during stress [41]. In addition, most *OfZAT*s contained only one exon, indicating their capacity to rapidly respond to environmental stresses. *Cis*-elements enable the binding of TFs and gene transcription [42]. Most *OfZATs* contained four types *cis*-elements associated with hormones, stress, development, and light responses (Figure S3), which suggested their potential functions in correlated reaction pathways.

Normally, the tissue-specific expression of genes implies their potential roles. For example, *AtERF102*, *AtERF103*, *AtERF104*, and *AtERF105*, which are predominantly expressed in root tissues, are cold stress regulator genes [43]. In this study, the differentially expressed *ZAT*s in *O. fragrans* tissues might crucially be involved in the plant growth and developmental processes. The genes involved in abiotic stress usually exhibited a differential expression profile in stress. For example, *AtZAT10* (*STZ*) responded to salt stress obviously and improved the resistance for salt stress [44]. *OfZAT8* and *OfZAT36* were strongly induced in salt stress and have a close phylogenetic relationship with *AtZAT10*, suggesting that the two genes are crucial for salt stress.

The expression of *OfZAT35* was upregulated under long-term cold treatment (Figure 8). Interestingly, *AtAZF2*, *AtZAT10*, and *AtZAT6*, which are cold stress responsive and regulators in *Arabidopsis*, were phylogenetically closely clustered with the candidate *OfZAT35* gene (Figure 1) [18,28]. Taken together, these results strongly suggested that *OfZAT35* might also play pivotal roles in cold stress response in *O. fragrans*. The *OfZAT35* protein was shown to be localized in the nucleus, which indicated its in-nucleus activity during cold stress response (Figure 8a). However, *OfZAT35* exhibited no transcriptional activation activity (Figure 8b). A similar phenomenon was also discovered in other ZFPs. For example, FaZAT10 exhibited no transcriptional activation activity in strawberry [17]. In Capsicum annuum, the full-length CAZFP1 protein had no transcriptional activation activity [45]. Previous research has revealed that ZFPs regulate gene transcription and expression by interacting with other ZFPs to bind to other DNA sequences [44,46,47]. Thus, the *OfZAT35* protein may interact with other proteins to regulate the expression of related genes and reduce cold tolerance. In addition, transiently transformed tobacco has been verified that overexpressed *OfZAT35* (Figure 9a) and showed a significantly higher REL level than in EV (*p* < 0.05) (Figure 9b). The REL is a decisive parameter for predicting the damage to the membrane system [48]. For example, the ectopic expression of *MdCDPK1a* could improve cold stress tolerance in *N. benthamiana* by reducing the REL value after cold stress exposure [49]. Similarly, the ectopic expression of *RmICE1* from *Rosa multiflora* enhanced cold stress tolerance in *N. benthamiana* and reduced the REL value after cold treatment [50]. Thus, the observed higher levels of REL indicated that *OfZAT35* overexpression reduced cold stress tolerance in *O. fragrans*. In addition, the activities of SOD, POD, and APX were all significantly increased, while that of CAT was dramatically decreased (Figure 9d). Correspondingly, the expression levels of *NbSOD*, *NbAPX*, and *NbCAT* were consistent with the activities of SOD, APX, and CAT (Figure 9c). Generally, the activities of antioxidant enzymes are induced when the plant is exposed to abiotic stress and could represent plant responses to adverse conditions [51]. The more significantly increased activities of SOD, POD, and APX in transiently transformed tobacco of *OfZAT35* may indicate that the overexpression of *OfZAT35* makes antioxidant enzymes more active to respond to cold stress. Furthermore, previous research has revealed that the *CAT* gene can reduce ROS levels to enhance cold tolerance [52,53]. Consequently, the decreased activity of CAT and expression level of *NbCAT* suggested that the transiently transformed tobacco of *OfZAT35* may accumulate an ROS level and exhibit more sensitivity to cold stress. *DREB*/*CBF* and *LEA* are essential genes that modulate cold stress, and the accumulation of their transcripts is positively correlated with enhanced cold tolerance [54,55,56]. The expression levels of *NbDREB3* and *NbLEA5* were significantly decreased (Figure 9c) and implied that *OfZAT35* is a likely negative regulator of cold stress tolerance in *O. fragrans*. 

## 4. Materials and Methods

### 4.1. Plant Materials and Treatments

Two-year-old cuttings of *O. fragrans* cv. ‘Rixianggui’ originated from Nanjing Forestry University in a previous study. Before application of abiotic treatments, seedlings with good development and consistent growth were selected and transferred to the growth chamber for one week. For cold treatment, the growth chamber temperature was adjusted to 4 °C, while other parameters were maintained [9]. During the cold treatment, we set seven timepoints (0, 3, 12, 24, 72, and 120 h during cold treatment, and after recovery 72 h) to collect the first two leaf pairs from seedlings as samples, and each timepoint set three biological replicates. For salt treatment, with other conditions maintained, the seedlings were planted in 1/2 Hoagland’s nutrient solution with 250 mM NaCl solution, and at four time points (0, 6, 24, and 72 h), we collected the first two leaf pairs from each biological replication as a sample for subsequent analyses. In addition, the seedlings of control group for salt stress were set by soaking in 1/2 Hoagland nutrient solution as previously described [32]. The samples were stored at −80 °C in a freezer.

### 4.2. Identification, Phylogenesis, Chromosomal Localization, Synteny, and Physicochemical Analysis of OfZATs

All protein-coding *OfZAT* gene sequences were obtained by genome-wide screening of *O. fragrans* sequence data [5]. The hidden Markov model (PF13912) was downloaded from the online database (http://pfam.xfam.org/ (accessed on 31 October 2022)) [16]. The HMMER 3.0 software was used to identify candidate *OfZAT* genes [57]. To examine the accuracy of conserved domains of the candidate *OfZAT* genes, the online tool CDD search (http://www.ncbi.nlm.nih.gov/ (accessed on 1 November 2022)) was used to verify the conserved domains. Moreover, physicochemical properties, such as pI and MW, were evaluated using an online tool (http://web.expasy.org/computepi (accessed on 31 October 2022)) [58]. Prediction of subcellular location was also performed using an online tool (https://wolfpsort.hgc.jp (accessed on 31 October 2022)) [59].

In total, 89 *ZAT* genes were retrieved from TAIR (https://www.arabidopsis.org/ (accessed on 10 November 2022)) and Phytozome13 (https://phytozome-next.jgi.doe.gov/ (accessed on 10 November 2022)), and their phylogenetic relationships assessed with the NJ method with 1000 bootstrap replications in MEGA 11.0 software [60].

The chromosomal locations of *OfZAT* genes were analyzed using TBtools based on the *O. fragrans* genome annotation files. The duplication events in ZAT family were determined using MCScanX (Multiple Collinearity Scan toolkit) program in TBtools, and the results were collected and then visualized using the advanced Circos program [61,62].

### 4.3. Gene Structure, Conserved Motif Compositions, and Cis-Element Analysis

TBtools was utilized to map the conserved domains of *ZAT* genes [63]. The motifs were forecasted through MEME tool (http://meme-suite.org/tools/meme (accessed on 7 November 2022)) [64]. A 2 kb genome sequence spanning the promoter regions of *OfZAT* genes was retrieved and screened for promoters using PlantCARE (https://bioinformatics.psb.ugent.be/webtools/plantcare/html (accessed on 7 November 2022)) [65]. The gene structure, motifs, and *cis*-elements of *ZAT*s were visualized in TBtools [63].

### 4.4. Expression Profiles of OfZATs in Tissues under Abiotic Stress

The FPKM values of *OfZAT*s in four tissues, including the root, stem, leaves, and flowers, were obtained from previously reported transcriptome data which have been published in NCBI (SRP143423) [4]. In addition, the expression patterns of *OfZATs* during cold and salt stress were also analyzed based on the unpublished transcriptome data, and the expression results were visualized with heat maps in TBtools [63].

### 4.5. RNA Extraction and qRT-PCR 

The extraction of total RNA and the synthesis of cDNA from the frozen leaf samples of *O. fragrans* were performed as in a previous study [9]. Primer 5.0 [66] was utilized to design specific primers for *OfZAT25*, *OfZAT26*, *OfZAT29*, and *OfZAT35* (Table S7). *OfRNA* was used to normalize the data analysis of qRT-PCR [7,67]. The reaction system of qRT-PCR was composed of 0.4 μL primer (forward and reverse primer, respectively), 5 μL SYBR, 0.2 μL ROX, 3 μL ddH_2_O, and 1 μL cDNA. The reaction condition was set as in a previous study [68]. 

The protocols of total RNA isolation from transiently transformed tobacco leaves and synthesis of cDNA were performed as in a previous study [9]. A semi-quantitative (sqRT-PCR) analysis was performed to verify the positive overexpression of *OfZAT35* in tobacco, and *Nbactin* was selected as reference gene (Table S7). The mix reaction solution of sqRT-PCR was composed of 1 μL cDNA, 1 μL primer, 10 μL 2 × Rapid taq, and 7 μL ddH_2_O. The primers of *NbSOD*, *NbCAT*, *NbP5CS*, *NbDREB3*, and *NbLEA5* for qRT-PCR are displayed in Table S8 [9]. Meanwhile, *Nbactin* was used as a normalizer for the qRT-PCR analysis. The qRT-PCR data were analyzed using the 2^−ΔΔCT^ method, and the results were statistically analyzed in SPSS 20.0 [7]. 

### 4.6. Subcellular Localization and Transcriptional Activation Activity of OfZAT35

Specific primers were designed for cloning the full-length CDS sequence of *OfZAT35* into the Super1300 vector between *Hind III* and *Kpn I* restriction sites to construct the 35S::*OfZAT35*-GFP fusion vector followed by a positive validation test (Table S9). The 35S::*OfZAT35*-GFP fusion vector and an EV were introduced into GV3101 (*Agrobacterium tumefaciens*), respectively, and incubated in Luria–Bertani culture with the following conditions: 28 °C, 200 rpm, 10 h. Then, *OfZAT35* and EV were injected into the leaves of 30-day-old tobacco plants, respectively. Transformed tobacco plants were incubated for 2 d in a growth room [9,32]. Finally, the fluorescent signals in the transgenic leaves were detected through an LSM710 microscope (Zeiss, Jena, Germany). Moreover, the whole ORF of *OfZAT35* was inserted into GAL4 DNA-binding domain between *Smal I* and *Sal I* restriction sites in pGBKT7 plasmid for transcriptional activation activity assay (primers are displayed in Table S8). The empty vector pGBKT7 (negative control) and fused vector pGBKT7-*OfZAT35* were introduced into AH109 yeast strain, respectively. Then, the yeast strain of pGBKT7-*OfZAT35* and pGBKT7 were respectively incubated in SD/Trp culture with the following conditions: 30 °C and 200 rpm until the value of OD600 reached 0.6. The yeast strains of pGBKT7-*OfZAT35* and pGBKT7 in 2 mL SD/Trp culture were collected into 100 μL ddH_2_O, and then diluted into different dilution multiples (10^0^, 10^−1^, 10^−2^, 10^−3^, and 10^−4^). The different dilution multiples solution of pGBKT7-*OfZAT35* and pGBKT7 was incubated in different nutrient deficient media (SD/-Trp, SD/-Trp-Ade, and SD/-Trp-Ade + X-α-gal medium) under 30 °C. 

Transiently transformed tobacco was generated as described for subcellular location. Transiently transformed tobacco was exposed to cold stress treatment at 4 °C in the growth chamber for 6 h. Samples were then collected and frozen by liquid nitrogen. The samples were stored at −80 °C in a freezer for RNA isolation and the determination of physiological properties.

### 4.7. Analyses of Physiological Parameters 

The REL value was determined to evaluate the degree of stress damage in the transgenic tobacco plants following a previously reported protocol [69]. Briefly, 0.2 g of shredded leaves was placed in 20 mL distilled water to measure the electrolyte leakage (C1) after 24 h, and the electrolyte leakage of pure distilled water (C0) was also determined. The shredded leaves in the pure distilled water were boiled for 30 min, and then the electrolyte leakage was determined (C2). The relative electrolyte leakage was calculated with the formula REL = (C1 − C0)/(C2 − C0) × 100%.

For enzyme activity assay, the crude enzyme solution was produced by 0.2 g leaf samples powder in 5 mL sodium phosphate buffer at pH 7.8. The assay for determining the activity of SOD referred to Beauchamp and Fridovich, while POD, CAT, and APX activities were quantified as previous in research with a slight modification [70,71,72]. The assay for POD activity was modified by adding 0.1 mL enzyme solution to the substrate solution and determining the reaction mixture at A470 nm. The assay for CAT activity was performed by adding 0.2 mL enzyme solution into substrate solution and determining the reaction mixture at A240 nm. The assay for APX activity was revised as adding 0.2 mL enzyme solution into substrate solution and determining the reaction mixture at A290 nm. The reaction mixture of POD, CAT, and APX was determined every 30 s during a total 180 s reaction time, respectively. 

## 5. Conclusions

In summary, this study identified 38 *OfZAT* genes, which could be classified into 5 subgroups based on phylogenetic relationships and sequence structures. The members of each subgroup contained similar motifs arranged in consistent patterns. In addition, 49 segmental and 5 tandem duplication events were detected among *OfZAT*s and might have contributed to the expansion of the *ZAT* gene family. The *ZAT* genes predominantly contained *cis*-elements associated with hormone, stress, light, and developmental processes. Numerous *OfZAT* genes showed tissue-specific expression patterns. Screening of the transcriptome data revealed two genes that could be induced by salt stress. Under cold stress, eight genes were strongly and differently expressed, of which *OfZAT35* showed a continuously increasing expression trend. *OfZAT35* is located in the nucleus, and it showed no transcriptional activation activity. The transient overexpression of *OfZAT35* in tobacco resulted in significantly higher REL values and downregulated the expression of positive to cold tolerance genes, such as *NbDREB3*, *NbLEA5*, and *NbCAT*, which suggested that *OfZAT35* is a negative regulator of cold tolerance in transgenic tobacco. Overall, this study not only expands the understanding of the *ZAT* gene family in *O. fragrans* but also provides a basis for the further functional evaluation of *OfZAT*s under cold stress.

## Data Availability

All data in this study could be found in the manuscript or supplemental materials.

## Supplementary Materials

The following are available online at https://www.mdpi.com/article/10.3390/plants12122346/s1, Figure S1: Synteny analysis of *ZAT* gene family across the *O. fragrans* genome, Figure S2: The phylogenetic classification and motif composition of 89 ZAT genes, Figure S3: The *cis*-element screening in the promoter regions (2-kb genome sequence) of *OfZATs*, Figure S4: The expression profiles of OfZATsin the salt stress treatment (S0 h, S6 h, S24 h, S72 h) and control (S0 h, CK6 h, CK24 h, CK72 h), Figure S5: The expression profiles of *OfZATs* during cold stress treatment, Table S1: The Physicochemical Characteristics of OfZATs, Table S2: The sequences of 15 motifs in 89 ZATs, Table S3: The sequences of 15 motifs in OfZATs, Table S4: The FPKM values of OfZATs in different *O. fragrans* tissues, Table S5: The FPKM valus of OfZATs in the salt stress treatment and control, Table S6: The FPKM values of OfZATs during cold stress treatment, Table S7: Primer sequences for qRT-PCR and sqRT-PCR, Table S8: Primer sequences for qRT-PCR, Table S9: Primer sequences used for *OfZAT35* amplification.
