# Supplementary material for: Genome-Wide Analysis of ZAT Gene Family in Osmanthus fragrans and the Function Exploration of OfZAT35 in Cold Stress
Source: Plants (Basel). 2023 Jun 16;12(12):2346. doi: 10.3390/plants12122346 (PMC10305554; doi:10.3390/plants12122346)
Supplement: Supplementary file 1 [file plants-12-02346-s001.zip › Figure S1 Syteny analysis.pdf]

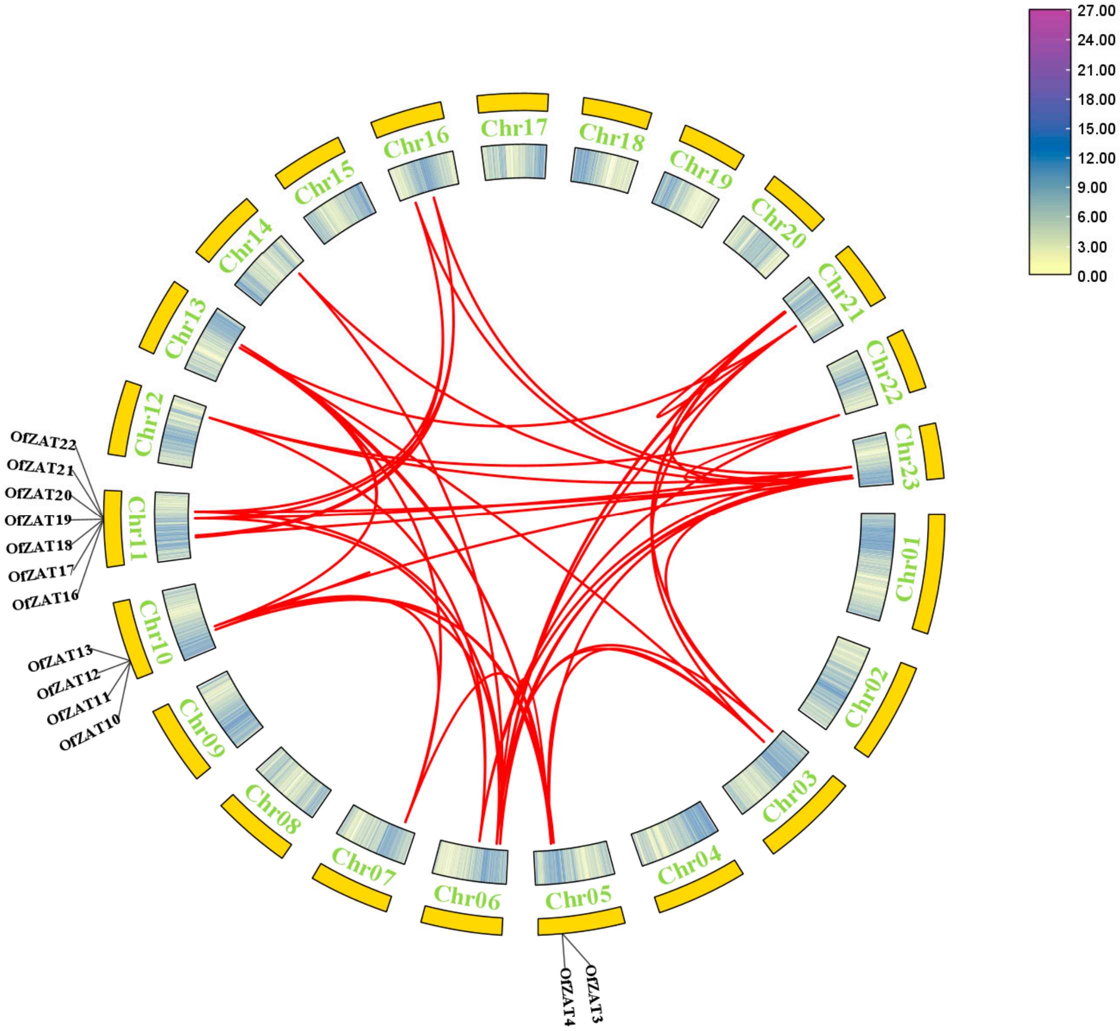

Figure S1. Synteny analysis of *ZAT* gene family across the *O. fragrans* genome. Syntenic linkages highlighted in red lines represent the 49 segmental duplications of *OfZAT* genes. The five tandemly duplicated *ZAT* genes are shown in the external yellow rectangles representing different chromosomes. The inner rectangles indicate gene density levels on chromosomes. The column legend on the right stand for the distribution density of ZAT gene in each chromosome.
