# Supplementary material for: Genome-Wide Analysis of ZAT Gene Family in Osmanthus fragrans and the Function Exploration of OfZAT35 in Cold Stress
Source: Plants (Basel). 2023 Jun 16;12(12):2346. doi: 10.3390/plants12122346 (PMC10305554; doi:10.3390/plants12122346)
Supplement: Supplementary file 1 [file plants-12-02346-s001.zip › Figure S2 motif of 89 ZAT genes.pdf]

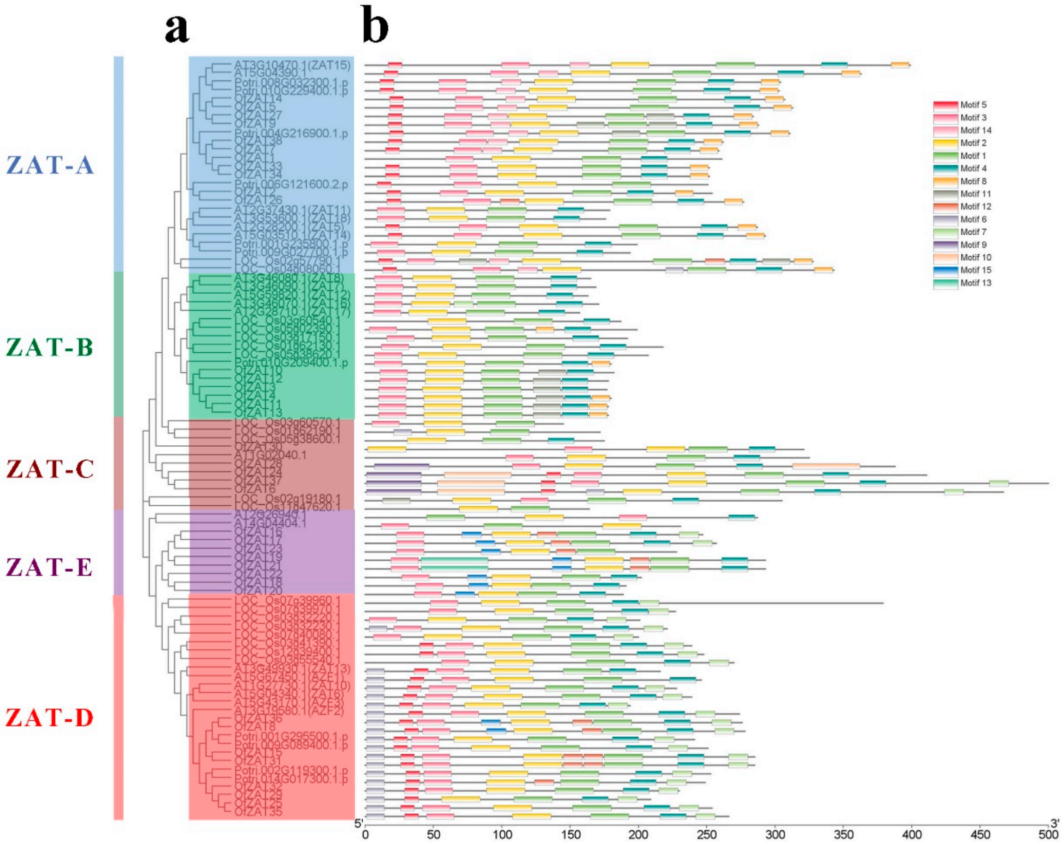

Figure S2. The phylogenetic classification and motif composition of 89 *ZAT* genes. (**a**) The phylogenetic tree of 89 *ZAT* gene from *O. fragrans*, *A. thaliana*, *O. sativa*, and *P. trichocarpa* showing different subgroups marked with different colors. (**b**) Conserved motifs in ZATs are highlighted in different colors.
