# Supplementary material for: Genome-Wide Analysis of ZAT Gene Family in Osmanthus fragrans and the Function Exploration of OfZAT35 in Cold Stress
Source: Plants (Basel). 2023 Jun 16;12(12):2346. doi: 10.3390/plants12122346 (PMC10305554; doi:10.3390/plants12122346)
Supplement: Supplementary file 1 [file plants-12-02346-s001.zip › Figure S3 cis-element analysis.pdf]

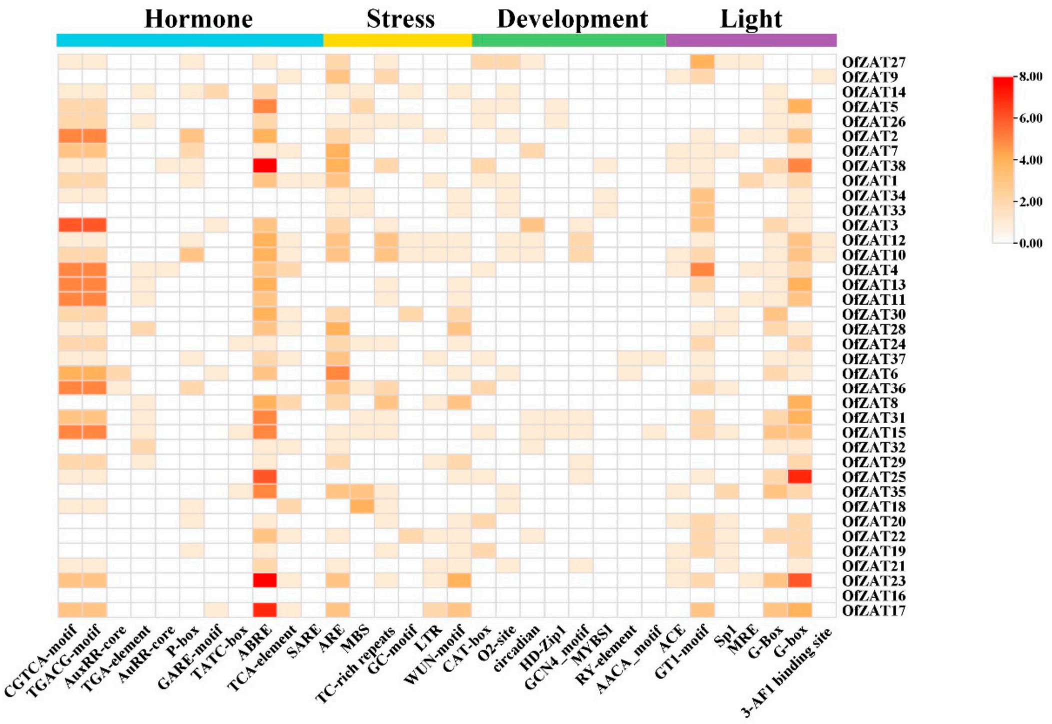

Figure S3. The *cis*-element screening in the promoter regions (2-kb genome sequence) of *OfZATs*. Most *ZAT* genes predominantly contained *cis*-elements associated with hormone, stress, development, and light responses. The color intensities indicate the number of *cis*-element in each gene.
