# Supplementary material for: Genome-Wide Analysis of ZAT Gene Family in Osmanthus fragrans and the Function Exploration of OfZAT35 in Cold Stress
Source: Plants (Basel). 2023 Jun 16;12(12):2346. doi: 10.3390/plants12122346 (PMC10305554; doi:10.3390/plants12122346)
Supplement: Supplementary file 1 [file plants-12-02346-s001.zip › Figure S4 salt stress heatmap.pdf]

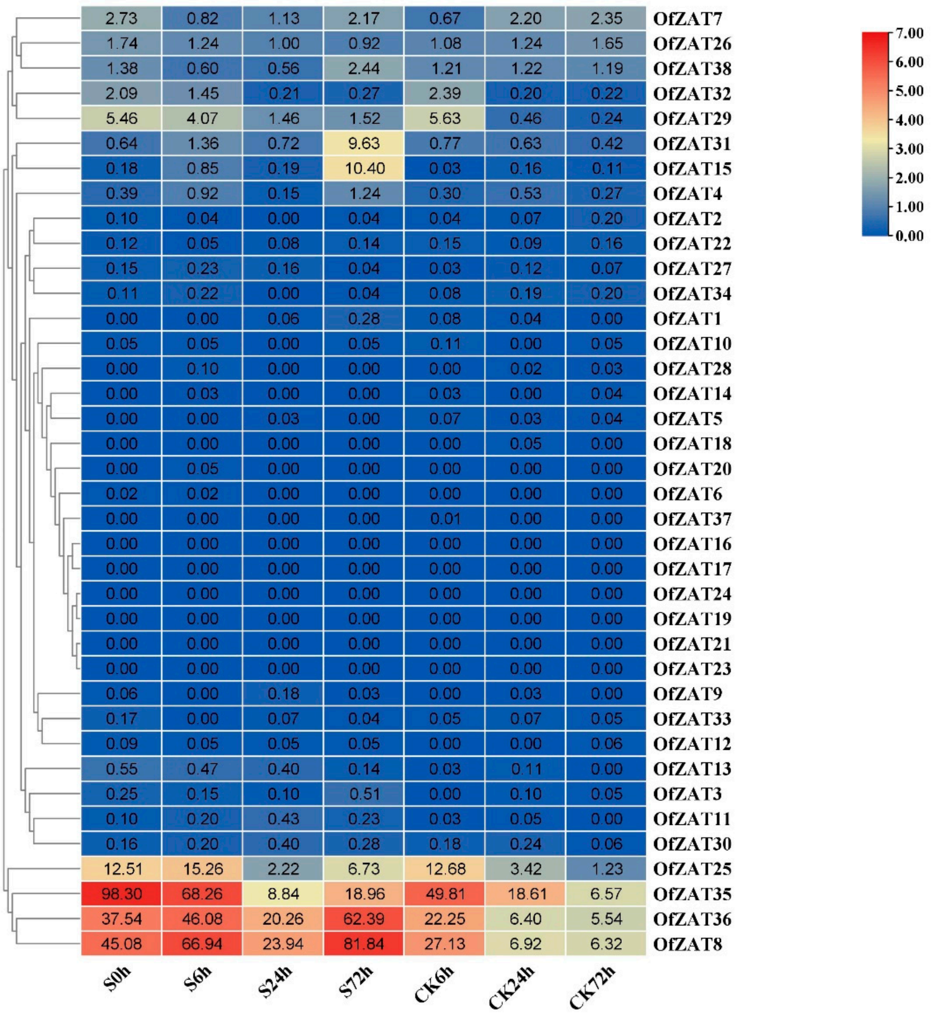

Figure S4. The expression profiles of OfZATsin the salt stress treatment (S0 h, S6 h, S24 h, S72 h) and control (S0 h, CK6 h, CK24 h, CK72 h). The hierarchically clustered heat map was constructed using the FPKM values converted to log_2 (FPKM values + 1). The original FPKM values are shown in the heat map. The column legend on the right stand for the color of log_2 (FPKM values + 1) in the heat map.
