# Supplementary material for: Genome-Wide Analysis of ZAT Gene Family in Osmanthus fragrans and the Function Exploration of OfZAT35 in Cold Stress
Source: Plants (Basel). 2023 Jun 16;12(12):2346. doi: 10.3390/plants12122346 (PMC10305554; doi:10.3390/plants12122346)
Supplement: Supplementary file 1 [file plants-12-02346-s001.zip › Figure S5 cold stress heatmap.pdf]

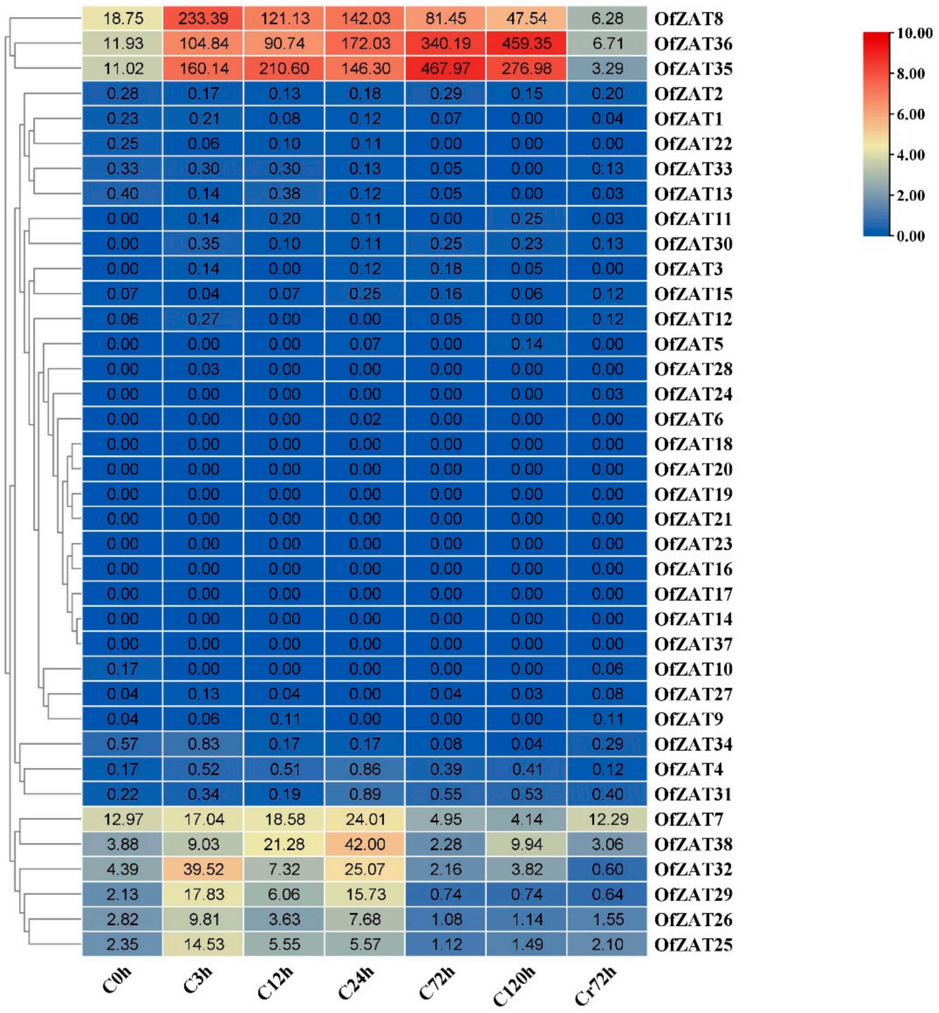

Figure S5. The expression profiles of *OfZATs* during cold stress treatment. The six periods during cold treatment were represent as C0 h, C3 h, C12 h, C24 h, C72 h and C120 h. Cr72 h represented recovering 72 h after cold treatment. The hierarchically clustered heat map was constructed using the FPKM values converted to $\log_2$ (FPKM values + 1). The original FPKM values are shown in the heat map. The column legend on the right stand for the color of $\log_2$ (FPKM values + 1) in the heat map.
